# Density Functional Theory (DFT) Study of Triphenylamine-Based Dyes for Their Use as Sensitizers in Molecular Photovoltaics

**DOI:** 10.3390/ijms13044418

**Published:** 2012-04-10

**Authors:** Jesús Baldenebro-López, José Castorena-González, Norma Flores-Holguín, Jorge Almaral-Sánchez, Daniel Glossman-Mitnik

**Affiliations:** 1 NANOCOSMOS Virtual Lab, Advanced Materials Research Center (CIMAV), Miguel de Cervantes 120, Complejo Industrial Chihuahua, Chihuahua 31190, México; E-Mails: jesus.baldenebro@cimav.edu.mx (J.B.-L.); norma.flores@cimav.edu.mx (N.F.-H.); 2 Faculty of Engineering Mochis, Autonomous University of Sinaloa, Prol. Ángel Flores y Fuente de Poseidón, S.N., Los Mochis, Sinaloa 81223, México; E-Mails: jhcg@uas.uasnet.mx (J.C.-G.); jalmaral@correo.uasnet.mx (J.A.-S.)

**Keywords:** molecular structure, absorption spectrum, polarizability, chemical reactivity, dipole moment, triphenylamine, dye sensitizers

## Abstract

In this work we studied three dyes which are proposed for potential photovoltaic applications and named Dye7, Dye7-2t and Dye7-3t. The Density Functional Theory (DFT) was utilized, using the M05-2X hybrid meta-GGA functional and the 6–31+G(d,p) basis set. This level of calculation was used to find the optimized molecular structure and to predict the main molecular vibrations, the absorption and emission spectra, the molecular orbitals energies, dipole moment, isotropic polarizability and the chemical reactivity parameters that arise from Conceptual DFT. Also, the p*K*_a_ values were calculated with the semi-empirical PM6 method.

## 1. Introduction

The world’s traditional energy sources (oil, natural gas and coal) have a finite life, and actual predictions indicate that alternative sources must provide an important contribution in the near future [1]. Solar energy is one of the most promising sources of energy in the future. The direct conversion of sunlight into electric energy using solar cells is particularly interesting because it has a lot of advantages over other methods, for example, it does not produce greenhouse gases nor nuclear byproducts [2]. The dye sensitized solar cells (DSSC) have attracted attention due to their efficiency, simple manufacturing and low cost [3–10]. In these DSSC, an organic sensitizer must be chemically absorbed on the porous surface of the nanocrystalline oxide. After absorbing a photon, the excited electron in the dye-sensitized molecule is transferred into the conduction band of nanocrystalline oxide, followed by a process in which the electron diffuses through the electrode. The sensitizer in this oxidized state is reduced to its normal state gaining electrons through a liquid electrolyte [11–13]. Nowadays, many research groups from all over the world actively participate to improve the efficiency of every single process involved in the DSSC [14–16]. The charge transfer efficiency from the dye molecule to the nanocrystalline oxide is extremely important in the solar cell design. Since Regal and Grätzel published their pioneer study [17], the understanding of the mechanism has required fundamental research about the diverse physical phenomena at nanometric scale [18].

Theoretical studies on physical and chemical properties of dye-sensitizers are very important to understand the relationship between the structure, properties and performance in order to design and synthesize new molecules for this purpose [19–23]. To be useful in DSSC a sensitizer must meet important requirements in its structure, such as: the electron-donating part [24,25], a unit to adjust the absorption spectrum [26] and the electron-acceptor part [27].

**Figure 1:**
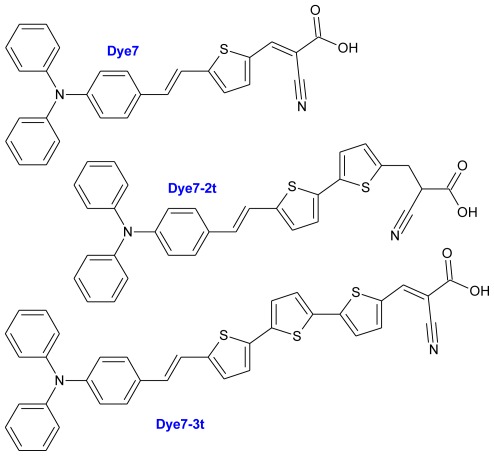
Sensitizer diagrams studied in this research (Dye7, Dye7-2t and Dye7-3t).

In the synthesis of new DSSC dyes, the triphenylamine based structures have been widely employed to build metal-free organic dyes and they have been successfully proven to show high conversion efficiency in DSSC devices [28–32] since they can both enhance the hole transporting ability of the materials and inhibit the aggregation of the dyes with their non-planar structure [33]. The tiophene molecule is included in the structure of the proposed systems due is one of the most frequently used π-spacer in organic dyes having a donor-(π-conjugated bridge)-acceptor (D-π-A) system in dye-sensitized solar cells (DSSCs) due to its high structural stability, ease of structural modification, excellent optical and electronic properties and controllable electrochemical behavior [34–39]; the π-conjugated derivates are generally efficient fluorophores, and as such, useful for the fabrication of nanobiosensors; they can be used as an attractive building block for Organic Molecular Materials [40]. The absorption spectra of the dye molecules can be easily tuned by changing the length of the oligothiophene bridge [24], this modification of the size of the thiophene chain not only will allow to evaluate the influence of these changes on the properties of absorption, but also on the emission spectra and band gap, among others. Moreover, the electron acceptor part has significant influences on the photovoltaic properties due to the fact that the excited electrons from the dye molecules are injected into the semiconductor film through this component [41]; a viable candidate that can efficiently act as a linker moiety is cyanoacrylic acid, since carboxylic acid binds strongly to the TiO2 surface through a bridging which is not easily removed by rinsing, and the presence of the cyano group enhances the electron-withdrawing nature of the linker moiety [39]. Based on this, we studied three sensitizers, which were named Dye7, Dye7-t2 and Dye7-t3 (Figure 1).

The aim of this work is to report the results of our research using molecular structure calculations and properties of the three dyes according to the density functional theory [42], developed by Walter Kohn in the 1960s. This method is implemented in the Gaussian 09W program package [43].

## 2. Results and Discussion

**Figure 2:**
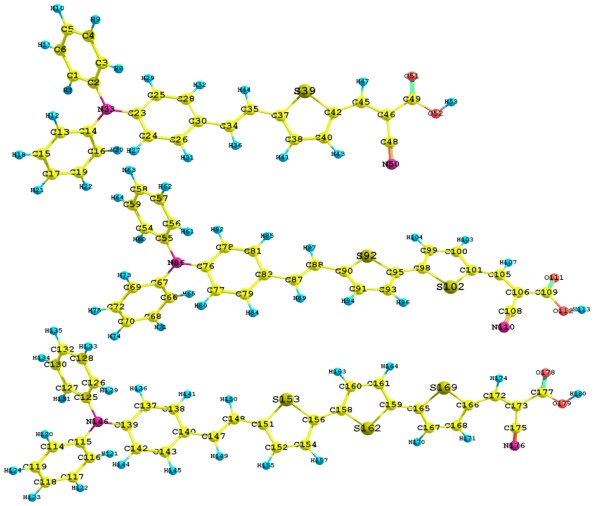
Optimized molecular structures of Dye7, Dye7-2t and Dye7-3t.

Once the molecular structures were proposed, the geometry optimization followed, which allowed the lowest energy configurations to be obtained, which are shown in Figure 2 including the numbers of atoms and symbols.

A selection of geometric parameters was made to clearly visualize how the geometric structures are similar regarding both conformation and geometric data when they are optimized using the M05-2X functional and the 6–31+G(d,p) basis set. Table 1 shows the selected values for bond length (Å), bond angles and dihedral angles (in degrees).

In IR spectra calculations there also appears to be a similarity in molecular structure due to the presence of vibrations with wave numbers close to each other. The O-H bond stretching vibration appears from 3836 cm^−1^ to 3843 cm^−1^. The vibration of the aromatic ring C-H bond is observed in the range from 3237 cm^−1^ to 3248 cm^−1^, and the range from 2406 cm^−1^ to 2416 cm^−1^ corresponds to the stretching of the triple bond C≡N. Other intense peaks that correspond to the stretching of the double bond carboxylic acid C=O and to the double bond between the thiophene molecule and the acid (C=C), appear in the ranges from 1835 cm^−1^ to 1842 cm^−1^ and from 1664 cm^−1^ to 1678 cm^−1^, respectively. The double bond C=C in the thiophene has a symmetric stretching vibration which presents from 1513 cm^−1^ to 1517 cm^−1^. The vibration of the bond C-N in the amine occurs in the range from 1380 cm^−1^ to 1391 cm^−1^. The corresponding vibration for the bending of the bond O-H produces a peak from 1201 cm^−1^ to 1223 cm^−1^. The bending of C-H in thiophene is present from 1086 cm^−1^ to 1093 cm^−1^. The out-of-plane bending vibration associated with the aromatic rings appears from 721 cm^−1^ to 726 cm^−1^. The C-S bond stretching vibration is shown in the wavenumbers ranging from 581 cm^−1^ to 598 cm^−1^.

**Figure 3:**
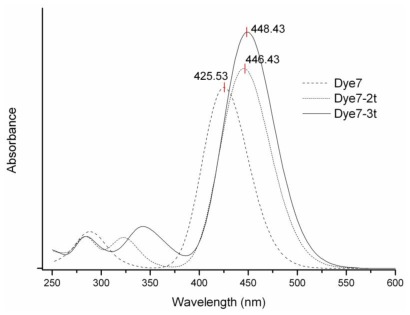
Ultraviolet-visible (UV-vis) spectra of Dye7, Dye7-2t and Dye7-3t.

Absorption spectra for the proposed dye molecules are shown in Figure 3. The calculated value of λmax is an important parameter, which indicates that these molecular systems should be considered for use as a functional material (as dye in this case) in a DSSC, the value of this parameter for Dye7, Dye7-2t and Dye7-3t meets the requirements established in the literature [44].

In all the UV-Vis spectra studied, the observed signal corresponds to the highest occupied molecular orbital (HOMO) to lowest unoccupied molecular orbital (LUMO) transition. Tables 2–4 show the results of TD-DFT calculations performed using the M05-2X functional and the 6–31+G(d,p) basis set; including the electronic excited states, the corresponding wavelengths (in nm), the energies (in eV), the oscillator strength (*f*) and the orbitals involved in the transitions.

**Figure 4:**
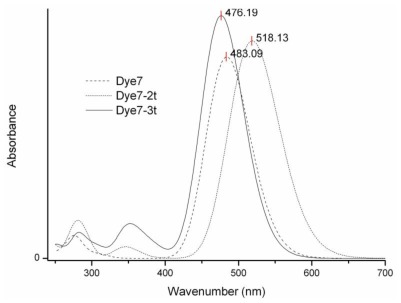
Fluorescence (fluo) spectra of sensitizers calculated using time-dependent DFT (TD-DFT) with the M05-2X/6–31+G(d,p) level.

To calculate the fluorescence spectra, the excited state optimization was carried out using the CIS/6-31+G(d,p) model [45]. The TD-DFT results are shown in Figure 4. The wavelength corresponding to the HOMO-LUMO transition is 483 nm for Dye7, 518 nm for Dye7-2t and 476 nm for Dye7-3t. These results indicate that the studied molecules have fluorescence in the visible region, and for this reason they constitute potential application in organic light emission diodes (OLEDs) [46].

**Figure 5:**
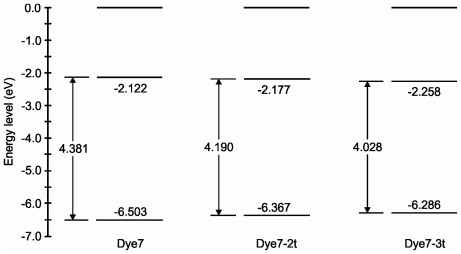
Molecular orbitals energy levels diagram.

In this work, a summary has been made based on the total dipole moment (for the ground state), the isotropic polarizability, and p*K*_a_ values (in water as solvent). This information is shown in Table 5. Figure 5 shows the HOMO-LUMO molecular orbitals’ energetic position. These results are of great importance, since they can be used during synthesis to determine the solubility and chemical reactivity of the molecule, and they can also be employed in organic electronics and photovoltaics, as reported in different studies [47–49].

The energy values of the orbitals indicate that these dyes can be used in a DSSC; this is a result of the sensitizer LUMO level and the conduction band of nanocrystalline oxides that are commonly used in such devices.

**Figure 6:**
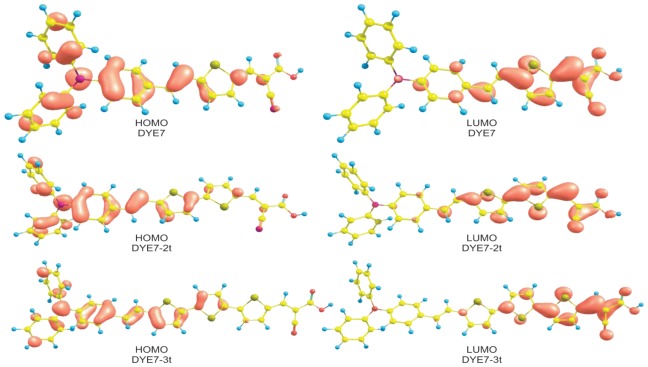
HOMO and LUMO orbitals of Dye 7, Dye7-2t and Dye7-3t calculated at the M05-2X/6–31G(d) level of theory.

The molecular orbitals HOMO and LUMO calculated at M05-2X/6–31+G(d,p) level of theory are shown in Figure 6. The reactive sites can be identified via orbital densities. The HOMO orbital density is located over the double bonds of the carbon chain and the nitrogen of the triphenylamine molecule, which indicates that in these sites an electrophilic attack can occur. Meanwhile, the density of the LUMO orbital is concentrated over the C-C single bonds; therefore, these are most likely sites for a nucleophilic attack.

The site for electrophilic attack will occur at atoms that produce a negative charge, and where the Fukui function *fk*^−^ is a maximal. The sites for nucleophilic attack will be those atoms that produce a positive charge and where the Fukui function *fk*^+^ is a maximal. The condensed Fukui function results for nucleophilic and electrophilic attacks were obtained with the AOMIX (a molecular analysis program) and are shown in Table 6.

As can be seen, these values confirm that the nitrogen atom in the triphenylamine molecule is the most likely site for the electrophilic attack, and the carbon atom that joins the thiophene chain to the acid is the site for the nucleophilic attack.

Chemical reactivity parameters such as ionization potential (I), electron affinity (A), electronegativity (χ), chemical hardness (η) and electrophilic index (ω) for the studied molecular systems (Table 7) were obtained by energy calculations (neutral and ionic state), taking into account the ground state geometry optimization.

## 3. Experimental Section

Computational calculations were carried out with the Gaussian 09W molecular modeling software obtaining the ground state molecular geometry for each dye. The strength constants and vibrational frequencies were determinated via analytic frequency calculations on stationary points obtained after geometry optimization. Both calculations were carried out at the same theory level. The chemical model utilized for the calculations was the 6–31+G(d,p) basis set [50–53] and the M05-2X hybrid meta-GGA functional [54], which have been proved to yield good results when modeling these kinds of structures.

Ultraviolet and fluorescence spectra in gas phase were carried out using Time-dependent density functional theory (TD-DFT) equations according to the method implemented in the molecular package Gaussian 09W [55–58]. The equations were solved for 20 excited states. The infrared (IR), ultraviolet-visible (UV-vis) and fluorescence (fluo) spectra were analyzed using the program SWizard [59]. The wavelength of maximum absorption and emission are shown for UV-vis and fluo spectra.

In this work we calculated the total dipole moment (μ) and the isotropic polarizability (α). The p*K*_a_ for the hydrogen atom attached to the oxygen atom was calculated using the MOPAC 2009 program [60]. In this program, the p*K*_a_ is calculated using the O-H distance calculated using PM6 [61]. Molecular dipole moment is an experimental measure of the charge distribution in a molecule. It is difficult to evaluate accurately the global electron distribution in a molecule because it involves all the multipoles. The polarizability contributes in a significant way to the understanding of the response of the system facing an external field.

The reactive sites can be identified using orbital densities. The condensed Fukui functions can also be used to determine the reactivity of each atom in the molecule. The corresponding condensed Fukui functions are given by *fk*^+^ = *qk*(*N* + 1) − *qk*(*N*) (nucleophilic attack), *fk*^−^ = *qk* (*N*) − *qk* (*N* − 1) (electrophilic attack) y *fk*^0^ = [*qk*(*N* + 1) − *qk*(*N −* 1)]/ 2 (radical attack), where *qk* is the effective Mulliken charge of atom *k* in the molecule. The condensed Fukui functions were evaluated with the AOMix molecular analysis program [62,63].

On the other hand, using the DFT framework makes it possible to find the chemical reactivity descriptors values, such as: electron affinity, ionization potential, electronegativity, hardness and electrophilic index. All these values were obtained using system energy calculations.

## 4. Conclusions

In this work, the molecular structure and properties of three molecules proposed as sensitizers in solar cells have been calculated. The applied methodology for this study is based on the density functional theory, using the M05-2x hybrid meta-GGA functional and the 6–31+G(d,p) basis set.

The molecular systems characterization includes the calculation of vibrations of functional groups, ultraviolet-visible and fluorescence spectra, total dipole moment, isotropic polarizability, p*K*_a_ values, molecular orbitals, chemical reactivity parameters and attack sites. The molecular orbitals energy indicates a smaller energy gap in the Dye7-3t sensitizer. For all three sensitizers, the LUMO orbital energies are similar, the difference being approximately 0.14 eV. Analyzing these data makes it possible to find potential applications for these dyes in photovoltaic devices.

## Figures and Tables

**Table 1:** Dye 7, Dye7-2t and Dye7-3t selected bond lengths (angstroms), bond angles and dihedral angles (degrees).

Dye7	Value	Dye7-2t	Value	Dye7-3t	Value
C2-N33	1.418	C55-N86	1.416	C125-N146	1.417
C14-N33	1.418	C67-N86	1.417	C115-N146	1.416
C23-N33	1.404	C76-N86	1.407	C139-N149	1.408
C2-C3	1.398	C55-C56	1.399	C125-C126	1.399
C15-C17	1.394	C70-C72	1.394	C118-C119	1.394
C30-C34	1.460	C83-C87	1.463	C140-C147	1.463
C35-C37	1.452	C88-C90	1.454	C148-C151	1.454
C42-C45	1.430	C101-C105	1.427	C166-C172	1.431
C2-N33-C23	120.3	C55-N86-C76	120.4	C125-N146-C139	120.2
C14-N33-C23	120.6	C67-N86-C76	120.1	C115-N146-C139	120.3
C30-C34-C35	126.6	C83-C87-C88	126.6	C140-C147-C148	126.3
C42-C45-C46	129.2	C101-C105-C106	130.2	C166-C172-C173	129.0
C2-N33-C23-C25	35.16	C55-N86-C76-C78	37.71	C125-N146-C139-C137	37.52
C23-N33-C14-C16	44.61	C66-C67-N86-C76	44.26	C116-C115-N146-C139	42.86
C45-C46-C49-O51	0.21	C105-C106-C109-O111	0.22	C172-C173-C177-O178	0.27

**Table 2:** Dye7 electronic excited states, showing wavelengths (nm), energies (eV), oscillator strength (*f*) and the orbitals involved in the transitions; calculated with Time-dependent density functional theory (TD-DFT) at M05-2X/6–31+G(d,p). Only excited states with *f* > 0.03 are shown.

Number	nm	eV	(*f*)	Assignment; H = HOMO, L = LUMO, L + 1 = LUMO + 1, *etc*.
1	425.5	2.91	1.6808	S H-0- > L + 0 (+73%) H-1- > L + 0 (8%)
2	320.5	3.87	0.0361	S H-1- > L + 0 (+55%) H-0- > L + 1 (19%)
3	301.4	4.11	0.0797	S H-0- > L + 2 (+42%) H-0- > L + 1 (+27%) H-1- > L + 0 (+10%) H-0- > L + 0 (+7%)
4	294.4	4.21	0.1563	S H-0- > L + 2 (+40%) H-0- > L + 1 (30%) H-0- > L + 0 (7%) H-1- > L + 0 (6%)
5	281.2	4.41	0.2058	S H-0- > L + 3 (+80%) H-1- > L + 3 (+9%)
6	266.4	4.65	0.0517	S H-6- > L + 0 (+44%) H-7- > L + 0 (14%) H-4- > L + 0 (+13%) H-1- > L + 1 (10%) H-0- > L + 4 (+6%)
7	259.4	4.78	0.0365	S H-0- > L + 5 (+40%) H-0- > L + 4 (15%) H-7- > L + 0 (11%)

**Table 3:** Dye7-2t electronic excited states, showing wavelengths (nm), energies (eV), oscillator strength (*f*) and the orbitals involved in the transitions; calculated with TD-DFT at M05-2X/6-31+G(d,p). Only excited states with *f* > 0.03 are shown.

Number	nm	eV	(*f*)	Assignment; H = HOMO, L = LUMO, L + 1 = LUMO + 1, *etc*.
1	446.4	2.78	1.8564	S H-0- > L + 0 (+67%) H-1- > L + 0 (22%) H-0- > L + 1 (+7%)
2	322.8	3.84	0.2720	S H-0- > L + 1 (+41%) H-0- > L + 0 (25%) H-1- > L + 0 (19%) H-1- > L + 1 (6%)
3	299.8	4.14	0.0368	S H-0- > L + 2 (+65%) H-0- > L + 3 (14%) H-1- > L + 2 (+8%)
4	286.8	4.32	0.0514	S H-1- > L + 1 (+36%) H-2- > L + 0 (22%) H-8- > L + 0 (14%) H-0- > L + 3 (11%) H-5- > L + 0 (+6%)
5	283.2	4.38	0.2221	S H-0- > L + 4 (+77%) H-1- > L + 4 (+17%)
6	258.6	4.79	0.0496	S H-0- > L + 5 (+28%) H-8- > L + 0 (+12%) H-5- > L + 0 (+11%) H-0- > L + 7 (7%)
7	248.9	4.98	0.0668	S H-0- > L + 6 (+42%) H-0- > L + 3 (12%) H-1- > L + 6 (+10%)
8	239.1	5.19	0.1066	S H-1- > L + 3(+15%) H-2- > L + 1(+11%) H-0- > L + 8(8%) H-0- > L + 7(+8%) H-9- > L + 0(8%) H-0- > L + 9(+8%)
9	231.1	5.36	0.0553	S H-4- > L + 0(+32%) H-4- > L + 1(+15%) H-0- > L + 7(+10%) H-7- > L + 0(10%) H-7- > L + 1(6%)

**Table 4:** Dye7-3t electronic excited states, showing wavelengths (nm), energies (eV), oscillator strength (*f*) and the orbitals involved in the transitions; calculated with TD-DFT at M05-2X/6-31+G(d,p). Only excited states with *f* > 0.03 are shown.

Number	nm	eV	(*f*)	Assignment; H = HOMO, L = LUMO, L + 1 = LUMO + 1, *etc*.
1	448.4	2.76	2.1948	S H-0- > L + 0 (+47%) H-1- > L + 0 (26%) H-0- > L + 1 (+12%)
2	364.3	3.40	0.1536	S H-0- > L + 1 (+49%) H-1- > L + 0 (+19%) H-0- > L + 2 (6%) H-2- > L + 0 (+6%)
3	339.3	3.65	0.3332	S H-0- > L + 0 (+47%) H-1- > L + 0 (+21%) H-0- > L + 1 (12%) H-2- > L + 0 (+9%) H-1- > L + 1 (+5%)
4	309.6	4.00	0.0758	S H-1- > L + 1 (+39%) H-0- > L + 2 (19%) H-2- > L + 0 (12%)
5	300.2	4.13	0.0462	S H-0- > L + 3 (+70%) H-1- > L + 3 (+13%) H-1- > L + 1 (+12%)
6	284.2	4.36	0.2165	S H-0- > L + 4 (+69%) H-1- > L + 4 (+18%)
7	278.2	4.46	0.0348	S H-0- > L + 2 (+29%) H-1- > L + 2 (27%) H-1- > L + 1 (+15%) H-0- > L + 1 (+6%)
8	255.8	4.85	0.0597	S H-10- > L + 0 (+32%) H-9- > L + 0 (13%) H-6- > L + 0 (8%)
9	243.6	5.09	0.0752	S H-0- > L + 10 (13%) H-2- > L + 1 (+12%) H-11- > L + 0 (+11%) H-0- > L + 5 (+10%) H-5- > L + 0 (+9%) H-6- > L + 0 (+6%)
10	234.0	5.30	0.0496	S H-2- > L + 2 (+13%) H-1- > L + 5 (+10%) H-0- > L + 11 (10%) H-9- > L + 0 (+6%)

**Table 5:** Dipole moment (μ), polarizability (α) and p*K*_a_ values.

Molecule	μ (Debye)	α (Bohr^3^)	p*K*_a_
Dye7	7.15	479.04	−0.17
Dye7-2t	6.42	578.60	−0.24
Dye-3t	7.13	684.24	−0.39

**Table 6:** Dye7, Dye7-2t and Dye7-3t nucleophilic and electrophilic attack sites.

Molecule	Site for Electrophilic Attack	Site for Nucleophilic Attack
Dye7	N33	C45
Dye7-2t	N86	C105
Dye7-3t	N146	C172

**Table 7:** Dye7, Dye7-2t and Dye7-3t chemical reactivity parameters calculated with M05-2X/6–31+G(d,p) using DFT descriptors.

Molecule	Conceptual DFT				

	I (eV)	A (eV)	χ (eV)	η (eV)	ω (eV)
Dye7	6.844	1.672	4.258	2.586	3.506
Dye7-2t	6.693	1.748	4.220	2.473	3.602
Dye7-3t	6.625	1.826	4.226	2.399	3.721
